# *In utero* particulate matter exposure in association with newborn mitochondrial ND4L_10550A>G_ heteroplasmy and its role in overweight during early childhood

**DOI:** 10.1186/s12940-022-00899-z

**Published:** 2022-09-19

**Authors:** Charlotte Cosemans, Congrong Wang, Rossella Alfano, Dries S. Martens, Hanne Sleurs, Yinthe Dockx, Kenneth Vanbrabant, Bram G. Janssen, Charlotte Vanpoucke, Wouter Lefebvre, Karen Smeets, Tim S. Nawrot, Michelle Plusquin

**Affiliations:** 1 Centre for Environmental Sciences, Hasselt University, Diepenbeek, Belgium; 2 Belgian Interregional Environment Agency, IRCEL-CELINE, Brussels, Belgium; 3 Flemish Institute for Technological Research, VITO, Mol, Belgium; 4 School of Public Health, Occupational & Environmental Medicine, Leuven University, Leuven, Belgium

**Keywords:** Mitochondria, SNP, Air pollution, DLMs, Childhood overweight

## Abstract

**Background:**

Mitochondria play an important role in the energy metabolism and are susceptible to environmental pollution. Prenatal air pollution exposure has been linked with childhood obesity. Placental mtDNA mutations have been associated with prenatal particulate matter exposure and MT-ND4L_10550A>G_ heteroplasmy has been associated with BMI in adults. Therefore, we hypothesized that in utero PM_2.5_ exposure is associated with cord blood MT-ND4L_10550A>G_ heteroplasmy and early life growth. In addition, the role of cord blood MT-ND4L_10550A>G_ heteroplasmy in overweight during early childhood is investigated.

**Methods:**

This study included 386 mother-newborn pairs. Outdoor PM_2.5_ concentrations were determined at the maternal residential address. Cord blood MT-ND4L_10550A>G_ heteroplasmy was determined using Droplet Digital PCR. Associations were explored using logistic regression models and distributed lag linear models. Mediation analysis was performed to quantify the effects of prenatal PM_2.5_ exposure on childhood overweight mediated by cord blood MT-ND4L_10550A>G_ heteroplasmy.

**Results:**

Prenatal PM_2.5_ exposure was positively associated with childhood overweight during the whole pregnancy (OR = 2.33; 95% CI: 1.20 to 4.51; *p* = 0.01), which was mainly driven by the second trimester. In addition, prenatal PM_2.5_ exposure was associated with cord blood MT-ND4L_10550A>G_ heteroplasmy from gestational week 9 – 13. The largest effect was observed in week 10, where a 5 µg/m^3^ increment in PM_2.5_ was linked with cord blood MT-ND4L_10550A>G_ heteroplasmy (OR = 0.93; 95% CI: 0.87 to 0.99). Cord blood MT-ND4L_10550A>G_ heteroplasmy was also linked with childhood overweight (OR = 3.04; 95% CI: 1.15 to 7.50; *p* = 0.02). The effect of prenatal PM_2.5_ exposure on childhood overweight was mainly direct (total effect OR = 1.18; 95% CI: 0.99 to 1.36; natural direct effect OR = 1.20; 95% CI: 1.01 to 1.36)) and was not mediated by cord blood MT-ND4L_10550A>G_ heteroplasmy.

**Conclusions:**

Cord blood MT-ND4L_10550A>G_ heteroplasmy was linked with childhood overweight. In addition, in utero exposure to PM_2.5_ during the first trimester of pregnancy was associated with cord blood MT-ND4L_10550A>G_ heteroplasmy in newborns. Our analysis did not reveal any mediation of cord blood MT-ND4L_10550A>G_ heteroplasmy in the association between PM_2.5_ exposure and childhood overweight.

**Supplementary Information:**

The online version contains supplementary material available at 10.1186/s12940-022-00899-z.

## Introduction

The World Health Organization (WHO) stated that exposure to ambient (outdoor) air pollution was estimated to cause 4.2 million premature deaths worldwide in 2016 [1]. Air pollution is a complex mix of gaseous and particulate components with high spatio-temporal heterogeneity [2]. The major components of particulate matter (PM) are acids, organic chemicals, including black carbon, metals, and soil or dust particles of varying diameters. Because of their small size, these particles can be inhaled deeply into the lungs and deposited in the alveoli. The smallest particles can directly reach the bloodstream [3]. Epidemiological and experimental studies have described the association between air pollution and adverse health effects in children and adults [4–6].

Air pollution has been associated with lower birth weight [7, 8], and reduced head circumference at birth [9]. Black carbon particles, an important component of PM, are even able to cross the placental barrier [10] which can explain the vulnerability of the foetus to maternal exposure to air pollution. Seo et al. [11] summarized several epidemiological studies investigating the association between prenatal air pollution and childhood obesity. Two cohort studies from the USA reported an association between prenatal PM_2.5_ exposure and obesity-linked aspects such as body mass index (BMI) at 2 – 9 years of age [12] and waist-to-hip ratio at four years of age [13]. However, the underlying mechanisms for this associations are poorly understood.

Mitochondria are responsible for energy production via oxidative phosphorylation, which is critical for normal cell functioning and foetal vitality. Dysfunctional mitochondria play a key role in metabolic disorders, such as type 2 diabetes and obesity [14]. Mitochondrial DNA (mtDNA) content can be an indicator of mitochondrial (dys)function [15]. Prenatal exposure to particles smaller than 10 microns (PM_10_) [16] and nitrogen dioxide (NO_2_) exposure [17] were associated with a decrease in placental mtDNA content. In addition, higher PM_2.5_ exposure was linked with lower mtDNA content in cord blood. In the recent study of Brunst et al. [18], mitochondrial mutational load was associated with prenatal PM exposure. They reported more nonsynonymous mitochondrial mutations in placental tissue with increased PM_2.5_ exposure, particularly in genes coding for nicotinamide adenine dinucleotide hydrogen (NADH) dehydrogenase and subunits of adenosine triphosphate (ATP) synthase.

Mitochondrial mutations can lead to the presence of multiple mtDNA sequences in a single cell, called heteroplasmy, which is often expressed as the percentage of the mutation-bearing sequence [19]. The association of mtDNA mutations and mitochondrial diseases have been studied before, but their role in common, complex disorders are not fully understood [19–22]. Given the important role of mitochondria in the energy metabolism and the fact that they are more prone to mutations, mitochondrial dysfunction contributes to cellular energy imbalance [23], leading to several metabolic disorders including obesity [23–25]. Studies also proved that mitochondrial dysfunction resulted in a dysregulation of lipid and glucose metabolism, which are characteristics of metabolic diseases [22]. Research on mitochondrial genetic variants in association with BMI in adults showed that the single nucleotide polymorphism (SNP) mitochondrial NADH dehydrogenase subunit 4L (MT-ND4L)_10550A>G_ was associated with a higher BMI [26]. MT-ND4L encodes one of the seven subunits of respiratory complex I (i.e., the mitochondrial NADH dehydrogenase subunit 4L). In humans, respiratory complex I is composed of 45 subunits. This particular subunit is responsible for the transfer of electrons from NADH to ubiquinone, the first step of the electron transport process, necessary to power the synthesis of ATP [26, 27]. Complex I is a source of damaging reactive oxygen species (ROS) and its dysfunction has been linked with mitochondrial disease, Parkinson’s disease, and aging [28]. Since this gene is important for energy production, it might play a role in the pathogenesis of childhood obesity. In this study, we investigate the association between in utero PM_2.5_ exposure and childhood overweight and a possible mediating role of cord blood MT-ND4L_10550A>G_ in this association.

## Materials and methods

### Study population and sample collection

This study population of 400 mother-newborn pairs was recruited between February 2010 and December 2015 as part of the ongoing prospective Environmental Influence on Aging in Early Life (ENVIR*ON*AGE) birth cohort, located in Flanders, Belgium (Supplementary Figure S1). Mother–child pairs were re-invited after 4 – 6 years for a follow-up visit. Details on recruitment of eligible mother-newborn pairs is described elsewhere [29] (details in supplemental information). After excluding 14 participants due to missing data (smoking during pregnancy, *n* = 1; maternal MT-ND4L_10550A>G_ heteroplasmy, *n* = 10) or failed measurements (maternal MT-ND4L_10550A>G_ heteroplasmy, *n* = 3), data was analysed for 386 mother-newborn pairs for the association between prenatal PM_2.5_ exposure and cord blood MT-ND4L_10550A>G_ heteroplasmy. For the association between prenatal PM_2.5_ exposure and childhood overweight and between cord blood MT-ND4L_10550A>G_ heteroplasmy and childhood overweight, three participants were excluded due to missing data (smoking during pregnancy, *n* = 1; childhood overweight, *n* = 2) and 29 participants did not participate in the follow-up study, i.e., data was analysed for 368 children. Finally, for the mediation analysis, an additional 13 participants were excluded due to missing data (maternal MT-ND4L_10550A>G_ heteroplasmy, *n* = 10) or failed measurements (maternal MT-ND4L_10550A>G_ heteroplasmy, *n* = 3), i.e., data was analysed for 355 mother–child pairs.

Umbilical cord blood samples were collected immediately after delivery in Vacutainer® Plus Plastic K2EDTA Tubes (BD, Franklin Lakes, NJ, USA). Maternal blood samples were collected 2–3 days after delivery in Vacutainer® Plus Plastic K2EDTA Tubes (BD). Samples were centrifuged at 3200 rpm for 15 min to retrieve buffy coats for DNA isolation. Samples were stored at -80 °C until further analysis.

Written informed consent was provided by all study participants in accordance with procedures approved by the Ethical Committee of Hasselt University and the East-Limburg Hospital. This study has been carried out according to the Helsinki declaration.

### Data collection

Mothers filled out a questionnaire at birth addressing their health status before and during pregnancy, including their smoking behaviour, education, and parity, as well as the newborn’s ethnicity and their residential address. In addition, the medical records provided information regarding pre-pregnancy BMI, maternal age, gestational age, newborn’s sex, and date of delivery. Mothers who smoked during pregnancy were coded “yes”, otherwise “no”. Maternal education was used as a proxy for socioeconomic status and was classified low (no diploma or primary school), middle (high school) or high (college or university). Parity was coded primi-, secundi- or multiparous. Ethnicity was classified as “European” when at least 2 of the newborn’s grandparents were European, otherwise “non-European”. Maternal pre-pregnancy BMI (kg/m^2^) was recorded at gestational weeks 7 – 9 by their gynaecologist. Season of delivery was categorized in “cold” (October 1^st^ – March 31^st^) or “warm” (April 1^st^ – September 30^th^) based on the date of delivery. At the follow-up visit, mothers filled out multiple questionnaires addressing general information about the lifestyle of the child and parents. Trained examiners performed the measurements of the clinical parameters, such as height and weight. WHO’s growth references during childhood were used to calculate the sex- and age-adjusted standard deviation (SD) BMI scores. Childhood overweight was defined as SD BMI scores being higher than the sex- and age-specific BMI cut-offs (Supplementary Table S1) according to the International Obesity Task Force (IOTF) [30].

### Prenatal air pollution exposure assessment

A high-resolution spatial–temporal interpolation method was used to model the outdoor PM_2.5_ concentrations (in µg/m^3^) based on the maternal residential address [31]. Address changes of mothers during pregnancy were taken into account. Briefly, land cover data from the satellite images from the CORINE land cover dataset was used to interpolate pollution data provided by the official fixed monitoring stations in the Flemish part of Belgium. In combination with a dispersion model that uses emissions from point sources (e.g., industrial sites) and line sources (e.g., road traffic ways), this model provides interpolated daily air pollution values on a dense, irregular high-resolution receptor point grid [32, 33]. The performance of the overall model was assessed by a leave-one-out cross-validation, which included 34 monitoring stations for PM_2.5_. The interpolation tool’s validation statistics explained more than 80% of the spatial–temporal variability in Flanders for PM_2.5_ [32, 34]. Further, the gestational exposure of this model was validated by the placental carbon load which depended on modeled outdoor PM_2.5_ concentration during pregnancy [10]. To explore potentially critical exposures during pregnancy, we calculated the exposures for specific time windows during pregnancy: the weekly average, the first trimester (i.e., date of conception until 13 weeks of pregnancy), the second trimester (i.e., 14 weeks until 26 weeks of pregnancy), the third trimester (i.e., 27 weeks pregnancy until delivery), and the entire pregnancy (i.e., gestational week 1 – 40). Ultrasound imaging data combined with the first day of the mother’s last menstrual period were used to estimate the date of conception [16].

### Mutation detection with droplet digital PCR

Total genomic DNA was extracted from buffy coats containing cord or peripheral maternal blood leukocytes using the QIAamp DNA mini kit (Qiagen, Venlo, the Netherlands) according to manufacturer’s instructions. DNA concentrations were assessed on a NanoDrop ND-1000 UV–Vis spectrometer (Thermo Scientific, Wilmington, DE) and stored at -80 °C until further analysis.

MtDNA mutational load was determined for MT-ND4L_10550A>G_ (i.e., rs28358280) using Droplet Digital PCR. The primer and probes were obtained from Bio-Rad (Hercules, CA, USA) (Assay ID: dHsaMDS169630047). Probes targeting the mutant allele were FAM labelled, while probes targeting the wild-type allele were HEX labelled. Droplet Digital PCR was performed according to manufacturer’s instructions, with some modifications as described below. Amplification was performed in a 20 µL reaction containing 2 × ddPCR Supermix for Probes (No dUTP), 900 nM primers and 250 nM of each probe, and 0.02 ng of DNA. Samples were partitioned into nanoliter-sized droplets using the QX200 Droplet Generator (Bio-Rad). After droplet generation, a PCR was performed with following cycling steps: enzyme activation at 95 °C for 10 min followed by 40 cycles of denaturation (95 °C, 30 s) and annealing (55 °C, 1 min), and finally enzyme deactivation at 98 °C for 10 min. To improve the droplet recovery to maximum (19,000 – 20,000 droplets), the PCR plate was incubated overnight at 4 °C [35]. Droplets were read on the QX200 Droplet Reader (Bio-Rad) and data were analysed using QuantaSoft™ Analysis Pro Software. Despite the variation in cord blood MT-ND4L_10550A>G_ heteroplasmy (Supplementary Figure S2), the measurements were dichotomized in order to improve their distribution. The presence of MT-ND4L_10550A>G_ heteroplasmy was classified as “yes” when the mutant allele was detected, independent of the level of this allele. Otherwise, MT-ND4L_10550A>G_ heteroplasmy was classified as “no”.

### Mitochondrial DNA content

DNA was isolated from cord blood buffy coat, containing leukocytes, using the QIAamp DNA mini kit (Qiagen). Leukocyte mtDNA content was measured by determining the ratio of the mitochondrial *ND1* gene to a single copy nuclear control gene (*RPLP0*) using the 7900HT Fast Real-Time PCR System (Applied Biosystems), as described elsewhere [16] (details in supplemental information).

### Statistical analysis

Data management and statistical analysis were done using R (version 4.1.2) and RStudio software (version 2021.09.0). For descriptive purposes, continuous variables (i.e., PM_2.5_ concentration, gestational age, maternal age, pre-pregnancy BMI, cord blood mtDNA content, and children’s age) were presented as means ± SD and categorical variables (i.e., cord blood MT-ND4L_10550A>G_ heteroplasmy, maternal MT-ND4L_10550A>G_ heteroplasmy, newborn’s sex, ethnicity, socioeconomic status, smoking behavior, parity, season of delivery, and childhood overweight) as numbers (frequency in percentage). All reported *p*-values were considered significant when lower than the nominal level of α = 0.05.

In a first step, the association between prenatal PM_2.5_ exposure and childhood overweight was evaluated with logistic regression models using the average trimester-specific residential PM_2.5_ exposures. Furthermore, the estimates for a 5 µg/m^3^ increment in PM_2.5_ were determined at each gestational week using distributed lag linear models (DLMs) [36]. These models were adjusted for a priori selected covariates, including gestational age, sex, ethnicity, maternal age, socioeconomic status, pre-pregnancy BMI, parity, smoking during pregnancy, child’s age at follow-up, and season of delivery. Second, the association between prenatal PM_2.5_ exposure and cord blood MT-ND4L_10550A>G_ heteroplasmy was evaluated with logistic regression models using the average trimester-specific residential PM_2.5_ exposures and at each gestational week using DLMs. These models were adjusted for a priori selected covariates based on previous studies [16, 37, 38], including gestational age, maternal age, pre-pregnancy BMI, newborn’s sex, ethnicity, socioeconomic status, smoking behavior, parity, and season of delivery. Trimester- and week-specific estimates are given as odds ratio (OR) per 5 µg/m^3^ increment in PM_2.5_ to indicate whether in utero PM_2.5_ exposure is associated with higher/lower odds of developing childhood overweight or cord blood MT-ND4L_10550A>G_ heteroplasmy.

The DLMs provide a flexible method to model the level of exposures while adjusting for lagged exposure values and thereby allows the identification of vulnerable exposure windows, which in turn provides hints on mechanisms through which exposure acts on fetal health [37, 39]. The exposure–response relationship and lag-response relationship are simultaneously involved in one model via the construction of a cross-basis accounting for both exposure structure and lag structure. We confirmed based on the models’ Akaike Information Criterion (AIC) that the exposure–response relationship was linear and specified for the lag structure a natural cubic spline with three inner knots equally spaced along the original lag scale (week 1 to week 40) based on a previous study [37]. The total degree of freedom (DF) of the cross-basis was five. Based on the DLM models, the cumulative association over the entire pregnancy as well as for each trimester was calculated as the incremental cumulative associations, respectively.

In a third step, the association between cord blood MT-ND4L_10550A>G_ heteroplasmy and childhood overweight was explored with logistic regression models, adjusted for gestational age, sex, ethnicity, maternal age, socioeconomic status, pre-pregnancy BMI, parity, smoking during pregnancy, cord blood mtDNA content, maternal MT-ND4L_10550A>G_ heteroplasmy, and child’s age at follow-up. Estimates were presented as OR to indicate whether cord blood MT-ND4L_10550A>G_ heteroplasmy is associated with higher/lower odds of developing childhood overweight. Lastly, a mediation analysis was performed using the imputation approach [40] to quantify the effects of prenatal PM_2.5_ exposure on childhood overweight mediated by cord blood MT-ND4L_10550A>G_ heteroplasmy. The total effect (TE) was decomposed into the natural direct effect (NDE) (i.e., unexplained by the mediator) and the natural indirect effect (NIE) (i.e., operating via the mediator). TE, NDE, and NIE are reported as OR and 95% CI. The outcome was modelled against prenatal PM_2.5_ exposure using a logistic regression model, adjusted for gestational age, maternal age, age of the child at follow-up, pre-pregnancy BMI, child’s sex, ethnicity, socioeconomic status, smoking behavior, parity, cord blood mtDNA content, maternal MT-ND4L_10550A>G_ heteroplasmy, and season of delivery. The 95% confidence intervals (CIs) were calculated by a bootstrap with 1000 replications.

## Results

### Population characteristics

The general characteristics of the study population (*n* = 386) are provided in Table 1. Gestation lasted on average 276 ± 11 days and newborns weighed 3370 ± 484 g. The majority of the newborns were girls (54.1%) of European descent (94%). The maternal age at the moment of delivery was 30.1 ± 4.2 years. Mothers had an average pre-pregnancy BMI of 24.3 ± 4.4, of which 21.0% were overweight and 11.4% were obese. Most of the mothers reported never having smoked (89.4%) and had a high educational level (64.8%). All births were equally distributed over the seasons and the majority were primiparous (55.7%). Cord blood mtDNA content was 1.2 ± 0.6. The residential prenatal exposure concentrations of PM_2.5_ by gestational time window are provided in Table 2. The average ambient PM_2.5_ concentration over the entire pregnancy was 14.02 ± 2.52 µg/m^3^, which is below average compared to several other European cohorts [41, 42]. The MT-ND4L_10550A>G_ variant was present in 8.5 and 13.2% of the cord blood and maternal blood samples, respectively. Cord blood MT-ND4L_10550A>G_ heteroplasmy was correlated with maternal MT-ND4L_10550A>G_ heteroplasmy (Pearson’s Chi-squared test: *p* < 0.0001; *R* = 0.6) and cord blood mtDNA content (*p* = 0.001; *R* = 0.2). Children at follow-up were on average 4.6 ± 0.4 years old, 13.6% of these children were overweight and 2.2% were obese.

**Table 1:** Study population characteristics. Childhood overweight was defined as WHO’s SD BMI scores being higher than the sex- and age-specific BMI cut-offs according to the International Obesity Task Force (IOTF) [30]

Characteristic	Mean ± SD or n (%)
***Maternal***^*a*^	
Age at delivery (years)	30.1 ± 4.2
Pre-pregnancy BMI	24.3 ± 4.4
Smoking during pregnancy (Yes)	41 (10.6)
Parity	
Primiparous	215 (55.7)
Secundiparous	135 (35.0)
Multiparous	36 (9.3)
Socioeconomic status	
Low	28 (7.2)
Middle	108 (28.0)
High	250 (64.8)
Season of delivery	
Cold (Oct 1^st^ – Mar 31^st^)	196 (50.8)
Warm (Apr 1^st^ – Sep 30^th^)	190 (49.2)
***Newborn*** ^*a*^	
Gestational age (days)	276 ± 11
Birth weight (g)	3370 ± 484
Sex (Female)	209 (54.1)
Ethnicity	
European	363 (94.0)
Non-European	23 (6.0)
***Child at follow-up*** ^*b*^	
Age (years)	4.6 ± 0.4
Childhood overweight (Yes)	50 (13.6)

^a^ Maternal and newborn’s characteristics: *n* = 386

^b^ Child’s characteristics: *n* = 368

**Table 2:** Residential prenatal exposure concentrations of PM_2.5_ (in µg/m^3^) by gestational time window

	**Mean ± SD**	**25**^**th**^** percentile**	**75**^**th**^** percentile**
Trimester 1	14.19 ± 5.21	10.73	17.86
Trimester 2	14.06 ± 5.06	9.72	17.63
Trimester 3	13.83 ± 5.42	9.44	17.39
Whole pregnancy	14.02 ± 2.52	12.03	15.87

### Association of prenatal PM_2.5_ exposure and childhood overweight

**Fig. 1:**
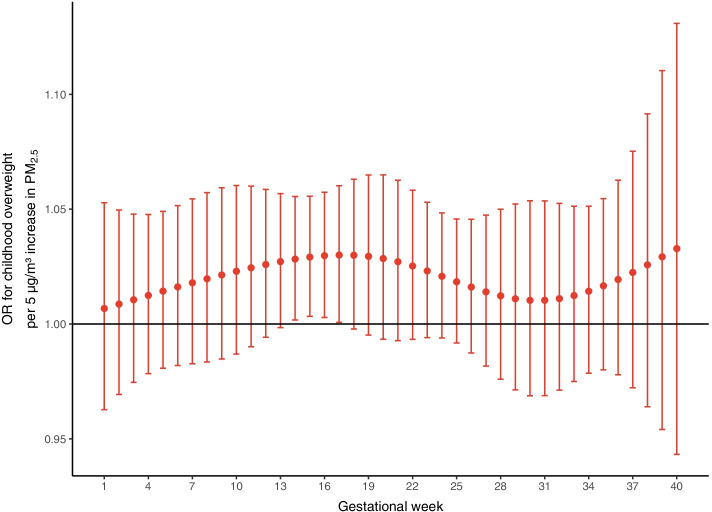
Odds ratio’s (OR) for childhood overweight in association with week-specific prenatal exposure to PM_2.5_. Week-specific estimates are given as odds ratio per 5 µg/m^3^ increment in PM_2.5_. Models were adjusted for gestational age, sex, ethnicity, maternal age, socioeconomic status, pre-pregnancy BMI, parity, smoking during pregnancy, age of the child at follow-up, and season of delivery. Error bars stand for the 95% CI for each weekly estimate

After adjustment for the preselected covariates, a 5 µg/m^3^ increment in prenatal PM_2.5_ exposure was positively associated with childhood overweight at four to six years of age (OR = 2.33; 95% CI: 1.20 to 4.51; *p* = 0.01; Supplementary Table S2). This association was mainly driven by the PM_2.5_ exposure in gestational week 14 – 17 (Fig. 1, Supplementary Table S3).

### Association of cord blood MT-ND4L_10550A>G_ heteroplasmy with prenatal PM_2.5_ exposure and childhood overweight

**Fig. 2:**
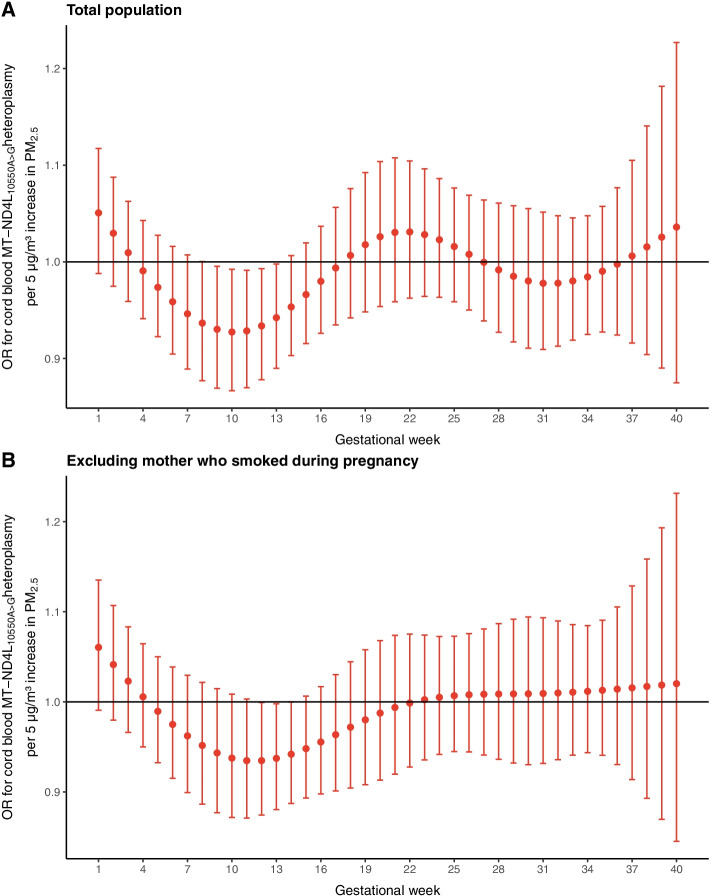
Odds ratio’s (OR) for cord blood MT-ND4L_10550A>G_ in association with week-specific prenatal exposure to PM_2.5_ for (**A**) the whole study population (*n* = 386) and (**B**) without mothers who smoked during pregnancy (*n* = 345). Week-specific estimates are given as odds ratio per 5 µg/m^3^ increment in PM_2.5_. Models were adjusted for gestational age, sex, ethnicity, maternal age, socioeconomic status, pre-pregnancy BMI, parity, season of delivery, smoking during pregnancy, cord blood mtDNA content, and maternal MT-ND4L_10550A>G_. Error bars stand for the 95% CI for each weekly estimate

Using the average trimester-specific residential PM_2.5_ exposures, we observed that a 5 µg/m^3^ increment in PM_2.5_ was associated with the cord blood MT-ND4L_10550A>G_ heteroplasmy (OR = 0.52; 95% CI: 0.26 to 1.03) in the first trimester with borderline significance (*p* = 0.06) (Supplementary Table S4). The week-specific DLM model showed a negative association between early pregnancy exposure to PM_2.5_ with cord blood MT-ND4L_10550A>G_. A 5 µg/m^3^ increment in PM_2.5_ was linked with cord blood MT-ND4L_10550A>G_ from gestational week 9 – 13. Week 10 exposure showed the largest effect (OR = 0.93; 95% CI: 0.87 to 0.99; Fig. 2A, Supplementary Table S5). In sensitivity analysis excluding mothers who smoked during pregnancy, cord blood MT-ND4L_10550A>G_ was associated with a 5 µg/m^3^ increment in PM_2.5_ only from gestational week 12 – 13 (Fig. 2B, Supplementary Table S6). Cord blood MT-ND4L_10550A>G_ and childhood overweight were significantly associated (OR = 3.04; 95% CI: 1.15 to 7.50; *p* = 0.02) after correction for the preselected covariates.

### Mediation analysis of MT-ND4L_10550A>G_ heteroplasmy in the association of prenatal PM_2.5_ exposure and childhood overweight

The mediation analysis showed that the effect of prenatal PM_2.5_ exposure on childhood overweight was mainly a direct effect (TE OR = 1.18; 95% CI: 0.99 to 1.36; NDE OR = 1.20; 95% CI: 1.01 to 1.36). There was no indirect effect via cord blood MT-ND4L_10550A>G_ heteroplasmy and confidence intervals included the unit (OR = 0.99; 95%CI: 0.96 to 1.02).

## Discussion

This study showed that in utero PM_2.5_ exposure was positively associated with being overweight at 4 – 6 years old, in particular exposure in gestational week 14 – 17. In addition, to our knowledge we were the first to link prenatal PM_2.5_ exposure with cord blood MT-ND4L_10550A>G_ heteroplasmy. Specifically, from gestational week 9 – 13, PM_2.5_ exposure was negatively associated with cord blood MT-ND4L_10550A>G_ heteroplasmy, while accounting for potential confounders. Furthermore, being overweight was positively associated with cord blood MT-ND4L_10550A>G_ heteroplasmy. MT-ND4L_10550A>G_ did not significantly mediate the association between PM_2.5_ exposure and being overweight in childhood.

Similar as in our study, several studies linked prenatal air pollution and childhood obesity [11]. Prenatal PM_2.5_ exposure was associated with BMI at 2 – 9 years of age [12] and waist-to-hip ratio at four years of age [13], which is consistent with our observations. Furthermore, in utero PM_2.5_ exposure has been associated with placental mtDNA mutations, especially among genes coding for NADH dehydrogenase (i.e., MT-ND5) and subunits of ATP synthase (i.e., MT-ATP6, MT-ATP8) [18]. A higher number of placental mitochondrial mutations in relation to PM_2.5_ exposure was observed mainly in mid- to late-pregnancy [18]. In contrast, our results linked prenatal PM_2.5_ exposure to lower cord blood MT-ND4L_10550A>G_ heteroplasmy in early pregnancy. A possible explanation for the negative association in our study between prenatal PM_2.5_ exposure and cord blood MT-ND4L_10550A>G_ from gestational week 9 – 13 may be due to the early protective mechanisms of the maternal liver and placenta and could reflect a shift in how it is adapted to accommodate the changing metabolizing demands of the foetus. The discrepancy between air pollution sensitivity time windows of the placenta (mid- to late-pregnancy) [18] and our results in cord blood (week 9 – 13) may lay in the ability of the placenta to protect the foetus from toxicants. The placenta has key functions including producing hormones or detoxifying enzymes and acts as a selective barrier [43]. However, the human placental barrier is not impenetrable for particles [10]. The maternal intra-placental circulation is not completely established until around 12 weeks of gestation, so substances must access the fetoplacental unit via diffusion through extracellular fluid or cell-to-cell transport. Therefore, the placenta may be less potent in the first trimester of pregnancy to protect the unborn from harmful substances [44]. In addition, compared to adults, the ability of the foetus to metabolize compounds is generally immature [45, 46]. As such, the human embryo is very vulnerable in the first trimester of the pregnancy. The foetus may be able to evade air pollution toxicity in part once the placenta is more operational. We showed that air pollution exposure during the 9^th^ until 13^th^ week of the pregnancy, slightly before the placenta is fully operational, is a very sensitive time window for cord blood MT-ND4L_10550A>G_ heteroplasmy.

Mitochondria are the main intracellular source as well as targets of ROS [16], which combined with the lack of protective histones and limited repair mechanisms, make them more prone to mutations, altered haplogroups or copy number [47]. Consequently, these disturbances can result in dysfunctionality of the electron transport chain which may even induce an increase in ROS generation [48]. Several components of ambient air pollutants, including PM_2.5_, ozone, and nitrogen oxides are able to generate ROS [49]. One important form of mutation that can be triggered by oxidative stress is a SNP, where one single nucleotide in the (mt)DNA sequence is altered in more than 1% of the population. The observed association between air pollution and cord blood MT-ND4L_10550A>G_ heteroplasmy is independent of the inherited maternal heteroplasmy. The MT-ND4L_10550A>G_ SNP in our study induces a codon change from ATA > ATG, both coding for the amino acid methionine, classifying it as a synonymous SNP which, opposite to nonsynonymous SNPs, does not lead to a change in amino acids [50]. For a long time, these synonymous SNPs were believed to be insignificant, since the primary sequence of the protein was maintained. However, several studies demonstrated that they can affect messenger RNA (mRNA) splicing [51], stability [52], and structure [53], as well as protein folding [54]. A study comparing synonymous and nonsynonymous SNPs found a similar likelihood and effect size of human disease associations for synonymous SNPs compared with nonsynonymous SNPs [55]. For example, a synonymous SNP in the *TNFSF15* gene (i.e., rs3810936) was linked with Crohn’s disease [56]. While most alterations due to synonymous SNPs are subtle, in combination with other SNPs and environmental factors they may become important [50].

MT-ND4L encodes one of the seven subunits of respiratory complex I (i.e., the mitochondrial NADH dehydrogenase subunit 4L), which is involved in metabolic pathways and oxidative phosphorylation [57]. A mitochondrial genome-wide association study (GWAS) investigating 984 mitochondrial SNPs to study genetic variants influencing BMI in adults, identified an association with MT-ND4L_10550A>G_ heteroplasmy [26]. This is consistent with our findings, as we also observed an association between cord blood MT-ND4L_10550A>G_ heteroplasmy and childhood overweight. These results suggest that this specific SNP is already linked with metabolic markers from early life onwards. In contrast, a study using single nucleotide protein genotyping reported a lack of relationship between mitochondrial heteroplasmy and childhood obesity [19]. Adverse prenatal exposures can promote permanent metabolic alterations in the foetus, causing the storage of excess calories, which in turn predisposes children to weight gain, leading to childhood overweight [58]. Animal studies reported that oxidative stress and mitochondrial damage in adipose tissue caused by air pollution increased both white and brown adipocytes differentiation, creating an energy imbalance in adipose tissue which leads to obesity and metabolic abnormalities [59, 60]. However, although we showed a positive association between cord blood MT-ND4L_10550A>G_ heteroplasmy and childhood overweight, our results did not indicate a causal mediation of cord blood MT-ND4L_10550A>G_ heteroplasmy in the association between prenatal PM_2.5_ exposure and childhood overweight. This may be due to the fact that the association between prenatal PM_2.5_ exposure and cord blood MT-ND4L_10550A>G_ heteroplasmy was observed in the first trimester of pregnancy, while the association between in utero PM_2.5_ exposure and childhood overweight was significant in the second trimester of pregnancy. In addition, it is also possible that this association is mediated by several SNPs or mitochondrial haplogroups, rather than only by MT-ND4L_10550A>G_. The link between other genetic variants in the MT-ND4L gene and human diseases have been described in several studies. In a study analysing 4220 mtDNA variants, MT-ND4L_10733C>A/T_ (i.e., rs28709356) was associated with Alzheimer’s disease [57]. The rare variant MT-ND4L_10652T>C_ has been associated with bipolar disorder and major depressive disorder [61]. In addition, the MT-ND4L_10609T>C_ and MT-ND4L_10663T>C_ variants were linked with Leber’s hereditary optic neuropathy [62]. Furthermore, higher mitochondrial heteroplasmy levels have been linked with a higher risk of all-cause dementia [63] and have been suggested as a possible biomarker for tumour progression [64]. In contrast, an experimental study reported that low-level mitochondrial heteroplasmy was linked with shorter murine lifespan, as well as impaired glucose metabolism [65]. To fully understand the specific role of MT-ND4L_10550A>G_ heteroplasmy in human health disorders, more research is required.

### Strengths and limitations

Our study has several strengths. We used a validated, high-resolution spatial–temporal interpolation method to assess daily PM_2.5_ exposures during pregnancy, which has been used in several studies [5, 66, 67] and was validated for gestational exposures and accumulation of nanoparticles in placental tissue [67]. To determine the MT-ND4L_10550A>G_ SNP, we used Droplet Digital PCR allowing the detection of low-level mutations by partitioning the PCR mixture into approximately 20,000 droplets, causing it to have superior sensitivity and accuracy compared to other methods [68]. We used DLMs to simultaneously represent linear exposure–response dependencies and delayed effects, allowing us to model weekly exposures of pregnancy to residential airborne particulate matter. Despite these strengths, we acknowledge some potential limitations in our study. The in utero time windows of PM_2.5_ estimates are different from the collecting time points of cord and maternal blood samples, which is at birth and 2 – 3 days after birth, respectively. Cord blood mutational load has only been established after delivery and therefore a time-dependent dynamic of mitochondrial load during gestation could not be determined and it does not necessarily reflect the status during the sensitive time windows. Despite the variation in cord blood MT-ND4L_10550A>G_ heteroplasmy (Supplementary Figure S2) and in contrast with other studies [18, 26], we considered MT-ND4L_10550A>G_ heteroplasmy as a binary variable due to the low VAF of MT-ND4L_10550A>G_ in the study population. In addition, it would be interesting to determine MT-ND4L_10550A>G_ heteroplasmy in placental tissue to compare the mutational load in both biological samples. Furthermore, this study focused on one variant in the *MT-ND4L* gene, but multiple other (mitochondrial) genes are involved in the energy metabolism and future studies should focus on studying other mitochondrial variants in relation to childhood overweight and obesity. PM_2.5_ exposures were modelled solely on the maternal residential address. However, other sources of exposure (i.e., while commuting, at work, etc.) can influence individual exposure to air pollutants. Nevertheless, the modelled residential PM_2.5_ and black carbon exposure estimates were correlated with internal black carbon load [67], which suggests that our model gives a proper estimation of individual exposures. Lastly, other environmental factors may also impact the presence of mtDNA mutations. However, we adjusted our models for several sociodemographic factors as well as environmental factors (i.e., season of delivery), which are linked with differential exposures [18].

## Conclusion

Exposure to particulate matter in the first trimester was positively associated with being overweight in early childhood. The neonate’s mitochondria are sensitive to environmental stressors during pregnancy, however little is known about the relationship of mitochondrial mutations in cord blood and prenatal air pollution exposure and their impact on overweight in children. To our knowledge, we were the first to observe an association between in utero PM_2.5_ exposure during the first trimester of pregnancy with cord blood MT-ND4L_10550A>G_ heteroplasmy in newborns, specifically at gestational week 9 – 13. Moreover, this variant was linked to overweight in early childhood. To further unravel the role of cord blood MT-ND4L_10550A>G_ heteroplasmy in childhood growth, more epidemiological studies are necessary.

## Supplementary Information

**Additional file 1: Study population**. Each year, the ENVIRONAGE birth cohort [29] recruits around 150 singleton births, making it the largest birth cohort with a prospective follow-up in Belgium. Mothers without planned caesarean section and able to fill out a Dutch language questionnaire are eligible for the cohort. We collect biological samples (e.g. placental tissue, cord blood, maternal blood) and have access to all medical records during and after pregnancy, including anthropometric and foetal ultrasound data in addition to lifestyle factors derived from questionnaires filled out after delivery. After birth, we follow these children throughout different stages of life, with a first follow-up visit at the age of 4 – 6 years. During follow-up, we collect biological tissues, lifestyle and medical data, and perform clinical and neurological measurements of both the child and the mother. **Measurement of mitochondrial DNA content.** DNA was isolated from cord blood buffy coat, containing leukocytes, using the QIAamp DNA mini kit (Qiagen). The relative amount of mtDNA was measured by determining the ratio of two mitochondrial gene copy numbers (MTF3212/R3319 and MT-ND1) to a single-copy nuclear control gene (RPLP0) using a real-time quantitative polymerase chain reaction (qPCR). qPCR reactions were carried out in triplicate on a 384-well plate on the 7900HT Fast Real-Time PCR System (Applied Biosystems) in a 10 μl volume containing: 5 μl Fast SYBR Green (Applied Biosystems) mastermix, 0.3 μl of forward and reverse primers (300 nM) and 1.9 μl RNAse-free water and 6 ng DNA diluted in 2.5 μl RNAse-free water. Primer sequences for mitochondrial genes are reported elsewhere [16]. Six interrun calibrators and no-template controls were included in each qPCR run. The thermal cycling profile for the three targets was 10 min at 95 °C for activation of the polymerase enzyme and initial denaturation, followed by 40 cycles of 15 s at 94 °C for denaturation and 70 s at 58 °C for annealing and extension. After thermal cycling, the raw data were collected and processed using SDS 2.3 software (Applied Biosystems). The cycle quantification (Cq) values were normalized relatively to the RPLP0 gene using qBase + software (Biogazelle) taking into account the run-to-run differences [69]. **Supplementary Table S1.** Sex- and age-specific BMI cut-offs according to the International Obesity Task Force (IOTF) [30]. **Supplementary Table S2.** The trimester-specific association between prenatal PM2.5 exposure and childhood overweight (*n* = 368). Models were adjusted for gestational age, sex, ethnicity, maternal age, socioeconomic status, pre-pregnancy BMI, parity, smoking during pregnancy, and child’s age at follow-up. Trimester-specific estimates of change are given as odds ratio per 5 µg/m³ increment in PM2.5. Childhood overweight was defined based on WHO’s SD BMI scores: > sex- and age-specific BMI cut-offs according to the International Obesity Task Force (IOTF) [30]. **Supplementary Table S3.** Difference in childhood overweight in association with week-specific prenatal exposure to PM2.5 (*n* = 368). Models were adjusted for gestational age, sex, ethnicity, maternal age, socioeconomic status, pre-pregnancy BMI, parity, smoking during pregnancy, and child’s age at follow-up. Week-specific estimates of change are given as odds ratio per 5 µg/m³ increment in PM2.5. **Supplementary Table S4.** The trimester-specific association between prenatal PM2.5 exposure and cord blood MT-ND4L10550A>G heteroplasmy (*n* = 386). Models were adjusted for gestational age, sex, ethnicity, maternal age, SES, pre-pregnancy BMI, parity, season of delivery, smoking during pregnancy, cord blood mtDNA content, and maternal MT-ND4L10550A>G. Trimester-specific estimates are given as odds ratio per 5 µg/m³ increment in PM2.5. **Supplementary Table S5.** Difference in cord blood MT-ND4L10550A>G heteroplasmy in association with week-specific prenatal exposure to PM2.5 (*n* = 386). Models were adjusted for gestational age, sex, ethnicity, maternal age, socioeconomic status, pre-pregnancy BMI, parity, season of delivery, smoking during pregnancy, cord blood mtDNA content, and maternal MT-ND4L10550A>G. Week-specific estimates are given as odds ratio per 5 µg/m³ increment in PM2.5. **Supplementary Table S6.** Difference in cord blood MT-ND4L10550A>G in association with week-specific prenatal exposure to PM2.5 without mothers who smoked during pregnancy (*n* = 345). Models were adjusted for gestational age, sex, ethnicity, maternal age, socioeconomic status, pre-pregnancy BMI, parity, season of delivery, cord blood mtDNA content, and maternal MT-ND4L10550A>G. Week-specific estimates are given as odds ratio per 5 µg/m³ increment in PM2.5. **Supplementary Figure S1.** Flowchart study population selection. The ENVIRONAGE birth cohort included 1993 mother-child pairs until March 2021. The study population (*n* = 400) was recruited between February 2010 and December 2015. The association between prenatal PM2.5 exposure and cord blood MT-ND4L10550A>G was analyzed for 386 mother-newborn pairs. The association between prenatal PM2.5 exposure or cord blood MT-ND4L10550A>G and childhood overweight was analyzed for 368 mother-newborn pairs. The mediation analysis was performed on 355 mother-child pairs. **Supplementary Figure S2.** Variation in cord blood MT-ND4L10550A>G heteroplasmy level (*n* = 400), ordered by study ID.

## Data Availability

The datasets used and/or analysed during the current study are available from the corresponding author on reasonable request.

## Abbreviations

AIC: Akaike information criterion
ATP: Adenosine triphosphate
BMI: Body mass index
CI: Confidence interval
DF: Degree of freedom
DLM: Distributed lag linear model
ENVIRONAGE: Environmental Influence on aging in early life
GWAS: Genome-wide association study
IOTF: International Obesity Task Force
mtDNA: Mitochondrial DNA
MT-ND4L: Mitochondrial NADH dehydrogenase subunit 4L
NADH: Nicotinamide adenine dicnucleotide hydrogen
NDE: Natural direct effect
NIE: Natural indirect effect
OR: Odds ratio
PM: Particulate matter
SD: Standard deviation
SNP: Single nucleotide polymorphism
TE: Total effect
WHO: World Health Organization
