# Supplementary material for: In utero particulate matter exposure in association with newborn mitochondrial ND4L10550A>G heteroplasmy and its role in overweight during early childhood
Source: Environ Health. 2022 Sep 19;21:88. doi: 10.1186/s12940-022-00899-z (PMC9484069; doi:10.1186/s12940-022-00899-z)
Supplement: Supplementary file 1 — Additional file 1: Study population. Each year, the ENVIRONAGE birth cohort [29] recruits around 150 singleton births, making it the largest birth cohort with a prospective follow-up in Belgium. Mothers without planned caesarean section and able to fill out a Dutch language questionnaire are eligible for the cohort. We collect biological samples (e.g. placental tissue, cord blood, maternal blood) and have access to all medical records during and after pregnancy, including anthropometric and foetal ultrasound data in addition to lifestyle factors derived from questionnaires filled out after delivery. After birth, we follow these children throughout different stages of life, with a first follow-up visit at the age of 4 – 6 years. During follow-up, we collect biological tissues, lifestyle and medical data, and perform clinical and neurological measurements of both the child and the mother. Measurement of mitochondrial DNA content. DNA was isolated from cord blood buffy coat, containing leukocytes, using the QIAamp DNA mini kit (Qiagen). The relative amount of mtDNA was measured by determining the ratio of two mitochondrial gene copy numbers (MTF3212/R3319 and MT-ND1) to a single-copy nuclear control gene (RPLP0) using a real-time quantitative polymerase chain reaction (qPCR). qPCR reactions were carried out in triplicate on a 384-well plate on the 7900HT Fast Real-Time PCR System (Applied Biosystems) in a 10 μl volume containing: 5 μl Fast SYBR Green (Applied Biosystems) mastermix, 0.3 μl of forward and reverse primers (300 nM) and 1.9 μl RNAse-free water and 6 ng DNA diluted in 2.5 μl RNAse-free water. Primer sequences for mitochondrial genes are reported elsewhere [16]. Six interrun calibrators and no-template controls were included in each qPCR run. The thermal cycling profile for the three targets was 10 min at 95 °C for activation of the polymerase enzyme and initial denaturation, followed by 40 cycles of 15 s at 94 °C for denaturation and 70 s at 58 °C for anneal [file 12940_2022_899_MOESM1_ESM.docx]

# SUPPLEMENTAL INFORMATION

## STUDY POPULATION

Each year, the ENVIR*ON*AGE birth cohort (29) recruits around 150 singleton births, making it the largest birth cohort with a prospective follow-up in Belgium. Mothers without planned caesarean section and able to fill out a Dutch language questionnaire are eligible for the cohort. We collect biological samples (e.g. placental tissue, cord blood, maternal blood) and have access to all medical records during and after pregnancy, including anthropometric and foetal ultrasound data in addition to lifestyle factors derived from questionnaires filled out after delivery. After birth, we follow these children throughout different stages of life, with a first follow-up visit at the age of 4 – 6 years. During follow-up, we collect biological tissues, lifestyle and medical data, and perform clinical and neurological measurements of both the child and the mother.

## MEASUREMENT OF MITOCHONDRIAL DNA CONTENT

DNA was isolated from cord blood buffy coat, containing leukocytes, using the QIAamp DNA mini kit (Qiagen). The relative amount of mtDNA was measured by determining the ratio of two mitochondrial gene copy numbers (MTF3212/R3319 and MT-ND1) to a single-copy nuclear control gene (RPLP0) using a real-time quantitative polymerase chain reaction (qPCR). qPCR reactions were carried out in triplicate on a 384-well plate on the 7900HT Fast Real-Time PCR System (Applied Biosystems) in a 10 μl volume containing: 5 μl Fast SYBR Green (Applied Biosystems) mastermix, 0.3 μl of forward and reverse primers (300 nM) and 1.9 μl RNAse-free water and 6 ng DNA diluted in 2.5 μl RNAse-free water. Primer sequences for mitochondrial genes are reported elsewhere (16). Six interrun calibrators and no-template controls were included in each qPCR run. The thermal cycling profile for the three targets was 10 min at 95 °C for activation of the polymerase enzyme and initial denaturation, followed by 40 cycles of 15 s at 94 °C for denaturation and 70 s at 58 °C for annealing and extension. After thermal cycling, the raw data were collected and processed using SDS 2.3 software (Applied Biosystems). The cycle quantification (Cq) values were normalized relatively to the RPLP0 gene using qBase + software (Biogazelle) taking into account the run-to-run differences (69).

## SUPPLEMENTARY TABLES

**Supplementary Table S1**: Sex- and age-specific BMI cut-offs according to the International Obesity Task Force (IOTF) (30).

| **Age (months)** | **Age (years)** | **Female** | | | | | | | | **Male** | | | | | | | |
| --- | --- | --- | --- | --- | --- | --- | --- | --- | --- | --- | --- | --- | --- | --- | --- | --- | --- |
|  |  | **BMI** | | | | | | | | **BMI** | | | | | | | |
|  |  | 16 | 17 | 18,5 | 23 | 25 | 27 | 30 | 35 | 16 | 17 | 18,5 | 23 | 25 | 27 | 30 | 35 |
| 48 | 4,0 | 12,9 | 13,5 | 14,3 | 16,5 | 17,4 | 18,1 | 19,2 | 20,6 | 13,0 | 13,7 | 14,5 | 16,7 | 17,5 | 18,3 | 19,2 | 20,6 |
| 49 | 4,1 | 12,8 | 13,4 | 14,3 | 16,5 | 17,3 | 18,1 | 19,2 | 20,6 | 13,0 | 13,6 | 14,5 | 16,7 | 17,5 | 18,2 | 19,2 | 20,6 |
| 50 | 4,2 | 12,8 | 13,4 | 14,3 | 16,5 | 17,3 | 18,1 | 19,2 | 20,6 | 13,0 | 13,6 | 14,5 | 16,7 | 17,5 | 18,2 | 19,2 | 20,6 |
| 51 | 4,3 | 12,8 | 13,4 | 14,2 | 16,5 | 17,3 | 18,1 | 19,1 | 20,6 | 13,0 | 13,6 | 14,4 | 16,6 | 17,5 | 18,2 | 19,2 | 20,6 |
| 52 | 4,3 | 12,8 | 13,4 | 14,2 | 16,4 | 17,3 | 18,1 | 19,1 | 20,6 | 13,0 | 13,6 | 14,4 | 16,6 | 17,5 | 18,2 | 19,2 | 20,6 |
| 53 | 4,4 | 12,7 | 13,3 | 14,2 | 16,4 | 17,3 | 18,1 | 19,1 | 20,7 | 12,9 | 13,6 | 14,4 | 16,6 | 17,4 | 18,2 | 19,2 | 20,6 |
| 54 | 4,5 | 12,7 | 13,3 | 14,2 | 16,4 | 17,3 | 18,1 | 19,1 | 20,7 | 12,9 | 13,5 | 14,4 | 16,6 | 17,4 | 18,2 | 19,2 | 20,6 |
| 55 | 4,6 | 12,7 | 13,3 | 14,1 | 16,4 | 17,3 | 18,1 | 19,2 | 20,7 | 12,9 | 13,5 | 14,4 | 16,6 | 17,4 | 18,2 | 19,2 | 20,6 |
| 56 | 4,7 | 12,7 | 13,3 | 14,1 | 16,4 | 17,3 | 18,1 | 19,2 | 20,7 | 12,9 | 13,5 | 14,3 | 16,6 | 17,4 | 18,2 | 19,2 | 20,7 |
| 57 | 4,8 | 12,7 | 13,3 | 14,1 | 16,4 | 17,2 | 18,1 | 19,2 | 20,7 | 12,9 | 13,5 | 14,3 | 16,6 | 17,4 | 18,2 | 19,2 | 20,7 |
| 58 | 4,8 | 12,6 | 13,2 | 14,1 | 16,4 | 17,2 | 18,1 | 19,2 | 20,8 | 12,8 | 13,4 | 14,3 | 16,5 | 17,4 | 18,2 | 19,2 | 20,7 |
| 59 | 4,9 | 12,6 | 13,2 | 14,1 | 16,3 | 17,2 | 18,1 | 19,2 | 20,8 | 12,8 | 13,4 | 14,3 | 16,5 | 17,4 | 18,2 | 19,3 | 20,8 |
| 60 | 5,0 | 12,6 | 13,2 | 14,0 | 16,3 | 17,2 | 18,1 | 19,2 | 20,8 | 12,8 | 13,4 | 14,3 | 16,5 | 17,4 | 18,2 | 19,3 | 20,8 |
| 61 | 5,1 | 12,6 | 13,2 | 14,0 | 16,3 | 17,2 | 18,1 | 19,2 | 20,9 | 12,8 | 13,4 | 14,2 | 16,5 | 17,4 | 18,2 | 19,3 | 20,8 |
| 62 | 5,2 | 12,5 | 13,1 | 14,0 | 16,3 | 17,2 | 18,1 | 19,2 | 20,9 | 12,8 | 13,4 | 14,2 | 16,5 | 17,4 | 18,2 | 19,3 | 20,9 |
| 63 | 5,3 | 12,5 | 13,1 | 14,0 | 16,3 | 17,2 | 18,1 | 19,3 | 21,0 | 12,7 | 13,3 | 14,2 | 16,5 | 17,4 | 18,2 | 19,4 | 21,0 |
| 64 | 5,3 | 12,5 | 13,1 | 14,0 | 16,3 | 17,2 | 18,1 | 19,3 | 21,0 | 12,7 | 13,3 | 14,2 | 16,5 | 17,4 | 18,2 | 19,4 | 21,0 |
| 65 | 5,4 | 12,5 | 13,1 | 14,0 | 16,3 | 17,2 | 18,1 | 19,3 | 21,1 | 12,7 | 13,3 | 14,2 | 16,5 | 17,4 | 18,3 | 19,4 | 21,1 |
| 66 | 5,5 | 12,5 | 13,1 | 13,9 | 16,3 | 17,3 | 18,1 | 19,4 | 21,2 | 12,7 | 13,3 | 14,2 | 16,5 | 17,4 | 18,3 | 19,5 | 21,2 |
| 67 | 5,6 | 12,4 | 13,0 | 13,9 | 16,3 | 17,3 | 18,2 | 19,4 | 21,2 | 12,6 | 13,3 | 14,1 | 16,5 | 17,4 | 18,3 | 19,5 | 21,2 |
| 68 | 5,7 | 12,4 | 13,0 | 13,9 | 16,3 | 17,3 | 18,2 | 19,4 | 21,3 | 12,6 | 13,2 | 14,1 | 16,5 | 17,5 | 18,3 | 19,6 | 21,3 |
| 69 | 5,8 | 12,4 | 13,0 | 13,9 | 16,3 | 17,3 | 18,2 | 19,5 | 21,4 | 12,6 | 13,2 | 14,1 | 16,5 | 17,5 | 18,4 | 19,6 | 21,4 |
| 70 | 5,8 | 12,4 | 13,0 | 13,9 | 16,3 | 17,3 | 18,2 | 19,5 | 21,4 | 12,6 | 13,2 | 14,1 | 16,5 | 17,5 | 18,4 | 19,7 | 21,5 |
| 71 | 5,9 | 12,4 | 13,0 | 13,9 | 16,3 | 17,3 | 18,3 | 19,6 | 21,5 | 12,6 | 13,2 | 14,1 | 16,5 | 17,5 | 18,4 | 19,7 | 21,6 |
| 72 | 6,0 | 12,3 | 13,0 | 13,9 | 16,3 | 17,3 | 18,3 | 19,6 | 21,6 | 12,5 | 13,2 | 14,1 | 16,5 | 17,5 | 18,5 | 19,8 | 21,7 |

**Supplementary Table S2: The trimester-specific association between prenatal PM_2.5_ exposure and childhood overweight (n = 368).** Models were adjusted for gestational age, sex, ethnicity, maternal age, socioeconomic status, pre-pregnancy BMI, parity, smoking during pregnancy, and child’s age at follow-up. Trimester-specific estimates of change are given as odds ratio per 5 µg/m³ increment in PM_2.5_. Childhood overweight was defined based on WHO’s SD BMI scores: > sex- and age-specific BMI cut-offs according to the International Obesity Task Force (IOTF) (30).

|  | Odds ratio (95% CI) | *p*-value |
| --- | --- | --- |
| Trimester 1 | 1.20 (0.82 to 1.75) | 0.35 |
| Trimester 2 | 1.45 (1.06 to 1.98) | 0.02 |
| Trimester 3 | 1.18 (0.81 to 1.70) | 0.39 |
| Whole pregnancy | 2.33 (1.20 to 4.51) | 0.01 |

**Supplementary Table S3:** **Difference in childhood overweight in association with week-specific prenatal exposure to PM_2.5_ (n = 368).** Models were adjusted for gestational age, sex, ethnicity, maternal age, socioeconomic status, pre-pregnancy BMI, parity, smoking during pregnancy, and child’s age at follow-up. Week-specific estimates of change are given as odds ratio per 5 µg/m³ increment in PM_2.5_.

|  | Odds ratio (95% CI) | p-value |  | Odds ratio (95% CI) | p-value |
| --- | --- | --- | --- | --- | --- |
| Week 1 | 1.01 (0.96 to 1.05) | 0.768 | **Week 21** | 1.03 (0.99 to 1.06) | 0.124 |
| Week 2 | 1.01 (0.97 to 1.05) | 0.671 | **Week 22** | 1.03 (0.99 to 1.06) | 0.123 |
| Week 3 | 1.01 (0.97 to 1.05) | 0.570 | **Week 23** | 1.02 (0.99 to 1.05) | 0.121 |
| Week 4 | 1.01 (0.98 to 1.05) | 0.479 | **Week 24** | 1.02 (0.99 to 1.05) | 0.132 |
| Week 5 | 1.01 (0.98 to 1.05) | 0.409 | **Week 25** | 1.02 (0.99 to 1.05) | 0.179 |
| Week 6 | 1.02 (0.98 to 1.05) | 0.360 | **Week 26** | 1.02 (0.99 to 1.05) | 0.275 |
| Week 7 | 1.02 (0.98 to 1.05) | 0.324 | **Week 27** | 1.01 (0.98 to 1.05) | 0.401 |
| Week 8 | 1.02 (0.98 to 1.06) | 0.292 | **Week 28** | 1.01 (0.98 to 1.05) | 0.514 |
| Week 9 | 1.02 (0.98 to 1.06) | 0.257 | **Week 29** | 1.01 (0.97 to 1.05) | 0.593 |
| Week 10 | 1.02 (0.99 to 1.06) | 0.216 | **Week 30** | 1.01 (0.97 to 1.05) | 0.633 |
| Week 11 | 1.02 (0.99 to 1.06) | 0.166 | **Week 31** | 1.01 (0.97 to 1.05) | 0.632 |
| Week 12 | 1.03 (0.99 to 1.06) | 0.111 | **Week 32** | 1.01 (0.97 to 1.05) | 0.593 |
| Week 13 | 1.03 (1.00 to 1.06) | 0.065 | **Week 33** | 1.01 (0.97 to 1.05) | 0.522 |
| Week 14 | 1.03 (1.00 to 1.06) | 0.037 | **Week 34** | 1.01 (0.98 to 1.05) | 0.439 |
| Week 15 | 1.03 (1.00 to 1.06) | 0.027 | **Week 35** | 1.02 (0.98 to 1.05) | 0.379 |
| Week 16 | 1.03 (1.00 to 1.06) | 0.031 | **Week 36** | 1.02 (0.98 to 1.06) | 0.366 |
| Week 17 | 1.03 (1.00 to 1.06) | 0.046 | **Week 37** | 1.02 (0.97 to 1.08) | 0.388 |
| Week 18 | 1.03 (1.00 to 1.06) | 0.069 | **Week 38** | 1.03 (0.96 to 1.09) | 0.423 |
| Week 19 | 1.03 (1.00 to 1.06) | 0.094 | **Week 39** | 1.03 (0.95 to 1.11) | 0.457 |
| Week 20 | 1.03 (0.99 to 1.06) | 0.114 | **Week 40** | 1.03 (0.94 to 1.13) | 0.486 |

**Supplementary Table S4: The trimester-specific association between prenatal PM_2.5_ exposure and cord blood MT-ND4L_10550A>G_ heteroplasmy (n = 386).** Models were adjusted for gestational age, sex, ethnicity, maternal age, SES, pre-pregnancy BMI, parity, season of delivery, smoking during pregnancy, cord blood mtDNA content, and maternal MT-ND4L_10550A>G_. Trimester-specific estimates are given as odds ratio per 5 µg/m³ increment in PM_2.5_.

|  | Odds ratio (95% CI) | *p*-value |
| --- | --- | --- |
| Trimester 1 | 0.52 (0.26 to 1.03) | 0.06 |
| Trimester 2 | 1.15 (0.56 to 2.36) | 0.70 |
| Trimester 3 | 0.74 (0.39 to 1.41) | 0.36 |
| Whole pregnancy | 0.56 (0.16 to 1.96) | 0.36 |

**Supplementary Table S5:** **Difference in cord blood MT-ND4L_10550A>G_ heteroplasmy in association with week-specific prenatal exposure to PM_2.5_ (n = 386).** Models were adjusted for gestational age, sex, ethnicity, maternal age, socioeconomic status, pre-pregnancy BMI, parity, season of delivery, smoking during pregnancy, cord blood mtDNA content, and maternal MT-ND4L_10550A>G_. Week-specific estimates are given as odds ratio per 5 µg/m³ increment in PM_2.5_.

|  | Odds ratio (95% CI) | p-value |  | Odds ratio (95% CI) | p-value |
| --- | --- | --- | --- | --- | --- |
| Week 1 | 1.05 (0.99 to 1.12) | 0.117 | **Week 21** | 1,03 (0.96 to 1.11) | 0.416 |
| Week 2 | 1.03 (0.97 to 1.09) | 0.297 | **Week 22** | 1,03 (0.96 to 1.10) | 0.386 |
| Week 3 | 1.01 (0.96 to 1.06) | 0.719 | **Week 23** | 1,03 (0.96 to 1.10) | 0.397 |
| Week 4 | 0.99 (0.94 to 1.04) | 0.720 | **Week 24** | 1,02 (0.96 to 1.09) | 0.460 |
| Week 5 | 0.97 (0.92 to 1.03) | 0.332 | **Week 25** | 1.02 (0.96 to 1.08) | 0.595 |
| Week 6 | 0.96 (0.90 to 1.02) | 0.155 | **Week 26** | 1.01 (0.95 to 1.07) | 0.796 |
| Week 7 | 0.95 (0.89 to 1.01) | 0.083 | **Week 27** | 1.00 (0.94 to 1.06) | 0.988 |
| Week 8 | 0.94 (0.88 to 1.00) | 0.052 | **Week 28** | 0.99 (0.93 to 1.06) | 0.808 |
| Week 9 | 0.93 (0.87 to 1.00) | 0.037 | **Week 29** | 0.99 (0.92 to 1.06) | 0.680 |
| Week 10 | 0.93 (0.87 to 0.99) | 0.030 | **Week 30** | 0.98 (0.91 to 1.06) | 0.595 |
| Week 11 | 0.93 (0.87 to 0.99) | 0.027 | **Week 31** | 0.98 (0.91 to 1.05) | 0.545 |
| Week 12 | 0.93 (0.88 to 0.99) | 0.029 | **Week 32** | 0.98 (0.91 to 1.05) | 0.526 |
| Week 13 | 0.94 (0.89 to 1.00) | 0.042 | **Week 33** | 0.98 (0.92 to 1.05) | 0.544 |
| Week 14 | 0.95 (0.90 to 1.01) | 0.085 | **Week 34** | 0.98 (0.92 to 1.05) | 0.621 |
| Week 15 | 0.97 (0.92 to 1.02) | 0.211 | **Week 35** | 0.99 (0.93 to 1.06) | 0.770 |
| Week 16 | 0.98 (0.93 to 1.04) | 0.481 | **Week 36** | 1.00 (0.92 to 1.08) | 0.950 |
| Week 17 | 0.99 (0.93 to 1.06) | 0.839 | **Week 37** | 1.01 (0.92 to 1.11) | 0.900 |
| Week 18 | 1.01 (0.94 to 1.08) | 0.846 | **Week 38** | 1.02 (0.90 to 1.14) | 0.796 |
| Week 19 | 1.02 (0.95 to 1.09) | 0.627 | **Week 39** | 1.03 (0.89 to 1.18) | 0.728 |
| Week 20 | 1.03 (0.95 to 1.10) | 0.491 | **Week 40** | 1.04 (0.87 to 1.23) | 0.682 |

**Supplementary Table S6:** **Difference in cord blood MT-ND4L_10550A>G_ in association with week-specific prenatal exposure to PM_2.5_ without mothers who smoked during pregnancy (n = 345).** Models were adjusted for gestational age, sex, ethnicity, maternal age, socioeconomic status, pre-pregnancy BMI, parity, season of delivery, cord blood mtDNA content, and maternal MT-ND4L_10550A>G_. Week-specific estimates are given as odds ratio per 5 µg/m³ increment in PM_2.5_.

|  | Odds ratio (95% CI) | p-value |  | Odds ratio (95% CI) | p-value |
| --- | --- | --- | --- | --- | --- |
| Week 1 | 1.06 (0.99 to 1.14) | 0.092 | **Week 21** | 0.99 (0.92 to 1.07) | 0.875 |
| Week 2 | 1.04 (0.98 to 1.11) | 0.194 | **Week 22** | 1.00 (0.93 to 1.08) | 0.973 |
| Week 3 | 1.02 (0.97 to 1.08) | 0.437 | **Week 23** | 1.00 (0.94 to 1.07) | 0.945 |
| Week 4 | 1.01 (0.95 to 1.06) | 0.847 | **Week 24** | 1.01 (0.94 to 1.07) | 0.879 |
| Week 5 | 0.99 (0.93 to 1.05) | 0.728 | **Week 25** | 1.01 (0.94 to 1.07) | 0.833 |
| Week 6 | 0.97 (0.92 to 1.04) | 0.433 | **Week 26** | 1.01 (0.94 to 1.08) | 0.812 |
| Week 7 | 0.96 (0.90 to 1.03) | 0.265 | **Week 27** | 1.01 (0.94 to 1.08) | 0.811 |
| Week 8 | 0.95 (0.89 to 1.02) | 0.172 | **Week 28** | 1.01 (0.94 to 1.09) | 0.819 |
| Week 9 | 0.94 (0.88 to 1.01) | 0.118 | **Week 29** | 1.01 (0.93 to 1.09) | 0.828 |
| Week 10 | 0.94 (0.87 to 1.01) | 0.084 | **Week 30** | 1.01 (0.93 to 1.09) | 0.830 |
| Week 11 | 0.93 (0.87 to 1.00) | 0.062 | **Week 31** | 1.01 (0.93 to 1.09) | 0.821 |
| Week 12 | 0.93 (0.87 to 1.00) | 0.049 | **Week 32** | 1.01 (0.94 to 1.09) | 0.800 |
| Week 13 | 0.94 (0.88 to 1.00) | 0.044 | **Week 33** | 1.01 (0.94 to 1.09) | 0.771 |
| Week 14 | 0.94 (0.89 to 1.00) | 0.051 | **Week 34** | 1.01 (0.94 to 1.08) | 0.744 |
| Week 15 | 0.95 (0.89 to 1.01) | 0.081 | **Week 35** | 1.01 (0.94 to 1.09) | 0.735 |
| Week 16 | 0.96 (0.90 to 1.02) | 0.153 | **Week 36** | 1.01 (0.93 to 1.11) | 0.749 |
| Week 17 | 0.96 (0.90 to 1.03) | 0.278 | **Week 37** | 1.02 (0.91 to 1.13) | 0.774 |
| Week 18 | 0.97 (0.90 to 1.04) | 0.439 | **Week 38** | 1.02 (0.89 to 1.16) | 0.799 |
| Week 19 | 0.98 (0.91 to 1.06) | 0.606 | **Week 39** | 1.02 (0.87 to 1.19) | 0.819 |
| Week 20 | 0.99 (0.91 to 1.07) | 0.754 | **Week 40** | 1.02 (0.85 to 1.23) | 0.835 |

## SUPPLEMENTARY FIGURES


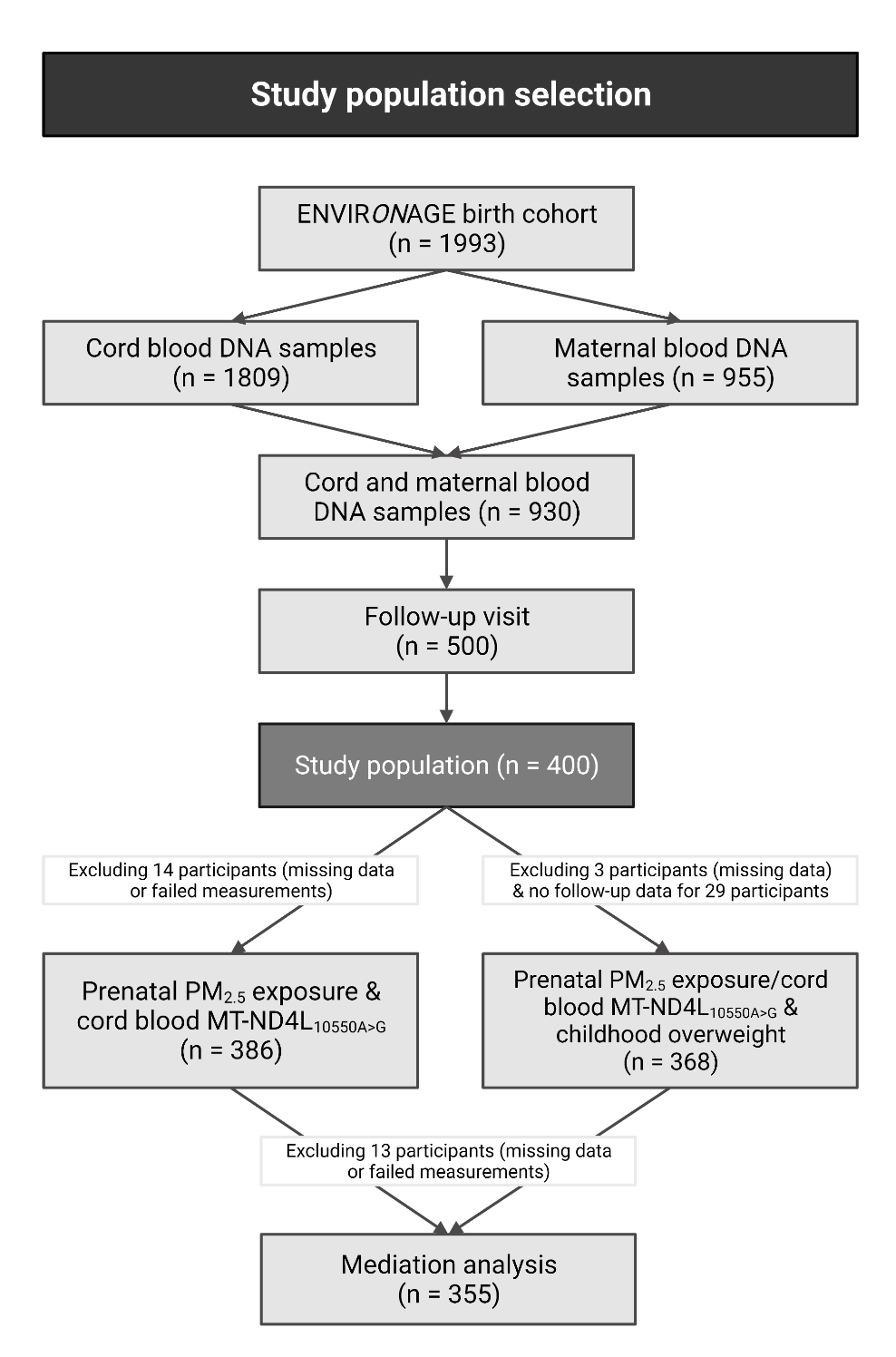


**Supplementary Figure S1: Flowchart study population selection.** The ENVIRONAGE birth cohort included 1993 mother-child pairs until March 2021. The study population (n = 400) was recruited between February 2010 and December 2015. The association between prenatal PM_2.5_ exposure and cord blood MT-ND4L_10550A>G_ was analyzed for 386 mother-newborn pairs. The association between prenatal PM_2.5_ exposure or cord blood MT-ND4L_10550A>G_ and childhood overweight was analyzed for 368 mother-newborn pairs. The mediation analysis was performed on 355 mother-child pairs.


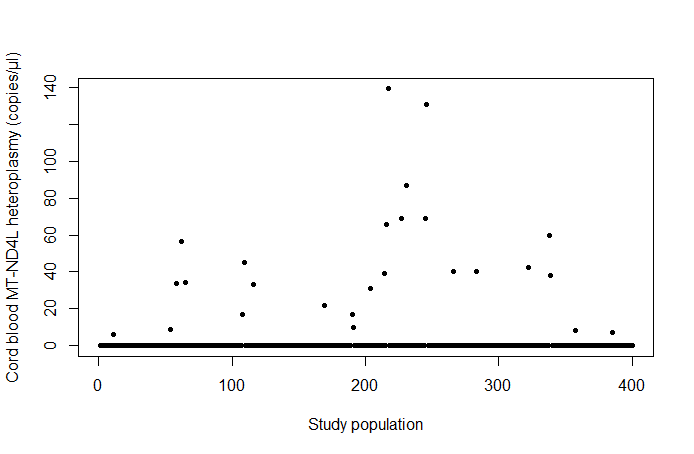


**Supplementary Figure S2: Variation in cord blood MT-ND4L_10550A>G_ heteroplasmy level (n = 400), ordered by study ID.**
